# Proximal Tracheal Mucoepidermoid Carcinoma Complicated by Early Anastomotic Stenosis—A Case Report

**DOI:** 10.1002/ccr3.73721

**Published:** 2026-10-10

**Authors:** Shivanjali Pandya, Gani Nuredini, Jahangir Ahmed, Khalid Ghufoor

**Affiliations:** ^1^ Barts and the London School of Medicine and Dentistry London UK; ^2^ The Royal London Hospital, Barts Health NHS Trust London UK

**Keywords:** airway disease, mucoepidermoid carcinoma, salivary‐type tumor, tracheal stenosis, tracheal tumor

## Abstract

Proximal tracheal mucoepidermoid carcinoma may remain occult despite prolonged upper‐airway symptoms and normal flexible nasoendoscopy. Stridor following tracheal resection should prompt assessment of the anastomosis, as early postoperative stenosis caused by A‐frame deformity and granulation tissue may be successfully managed with combined laser therapy, balloon dilatation, and intralesional corticosteroid injection.

## 1 Introduction

Primary malignant tumors of the trachea are rare. Mucoepidermoid carcinoma (MEC) is an uncommon epithelial neoplasm characterized by a mixture of mucous, intermediate, and epidermoid cells. Although MEC most frequently arises within the major and minor salivary glands [1], where it constitutes approximately 5%–15% of salivary gland malignancies [2], it has been described at several non‐salivary sites, including the bronchial tree, breast, and thyroid [3, 4, 5].

Tracheal MEC is exceptionally uncommon, particularly in the cervical trachea, with only a small number of isolated case reports or small series documented in the literature [3, 6]. Its indolent and nonspecific presentation may resemble benign airway disease (e.g., asthma or chronic bronchitis) and delay diagnosis [6]. Staging, feasibility of complete surgical resection, and histological grade are important determinants of management and prognosis [6].

We report a case of cervical tracheal MEC presenting after 4 years of upper‐airway symptoms despite normal flexible nasoendoscopy. The report focuses particularly on the development and recognition of early postoperative tracheal stenosis and its successful multimodal endoscopic management following curative tracheal resection.

## 2 Case Presentation

### 2.1 Patient Information and Clinical Findings

A 63‐year‐old woman was referred to the rapid‐access head and neck clinic after a tracheal lesion was identified on imaging performed during evaluation of a palpable neck mass.

She described a 4‐year history of frequent throat clearing and persistent globus pharyngeus that disturbed her sleep, together with intermittent exertional stridor. Despite these symptoms, she remained physically active. Relevant medical history included osteoarthritis, gastro‐esophageal reflux disease, and prior radioiodine ablation for a multinodular goiter in 1998. She was a lifelong non‐smoker with reported moderate alcohol intake.

Clinical examination demonstrated a firm midline neck lump immediately below the level of the cricoid, without overlying skin change or cervical lymphadenopathy. Flexible nasoendoscopy was unremarkable. The clinical course from symptom onset through definitive treatment and management of the postoperative stenosis is summarized in Table 1.

**TABLE 1:** Clinical timeline from symptom onset to postoperative endoscopic management.

Time	Clinical event
2019 (four years prior to diagnosis)	Onset of frequent throat‐clearing and persistent globus sensation, followed by intermittent exertional stridor.
September 2023 — Initial referral to Head and Neck centre	Assessment of a palpable neck lump. Flexible nasoendoscopy was normal.
October 2023 — Diagnostic imaging	Ultrasound suggested benign multinodular goiter. Cross‐sectional imaging identified a 10 × 10 × 18 mm intraluminal lesion at the second and third tracheal rings.
October 2023 — Initial endoscopic assessment	Microlaryngobronchoscopy showed a broad‐based lesion obstructing approximately 70% of the tracheal lumen. Partial CO_2_ laser debulking and biopsy were performed.
November 2023 — Definitive treatment	Total thyroidectomy, segmental tracheal resection and reconstruction with a rotational muscle flap.
Definitive histopathology	Resection histology confirmed completely excised tracheal MEC with clear margins and no cartilage invasion.
Postoperative day 3	Transient hypocalcaemia resolved.
Postoperative day 5	Acute dyspnoea, left‐sided neck swelling, and localized surgical emphysema. Symptoms resolved following partial wound opening.
Postoperative day 15	Discharged from hospital.
December 2023 / Postoperative day 25	Re‐presented with positional stridor and narrowing at the operative site, initially treated with systemic corticosteroids and nebulised adrenaline.
January 2024 / Six weeks postoperatively	Bronchoscopy confirmed significant tracheal stenosis with deformity. Biopsy demonstrated postoperative granulation tissue, fibrosis and inflammation, with no definite recurrent MEC. Laser therapy, balloon dilatation, and intralesional corticosteroid injection were performed.
Eight weeks postoperatively, two weeks after endoscopic intervention	Complete resolution of symptoms was reported.

### 2.2 Patient Timeline

See Table 1.

### 2.3 Diagnostic Assessment

Neck ultrasound suggested a benign multinodular goiter corresponding to the palpable neck swelling. However, subsequent cross‐sectional imaging demonstrated an intraluminal tracheal lesion measuring 10 × 10 × 18 mm at the level of the second and third tracheal rings. This lesion appeared confined to the tracheal lumen, without radiological evidence of extraluminal extension or regional lymphadenopathy (Figure 1).

**FIGURE 1:**
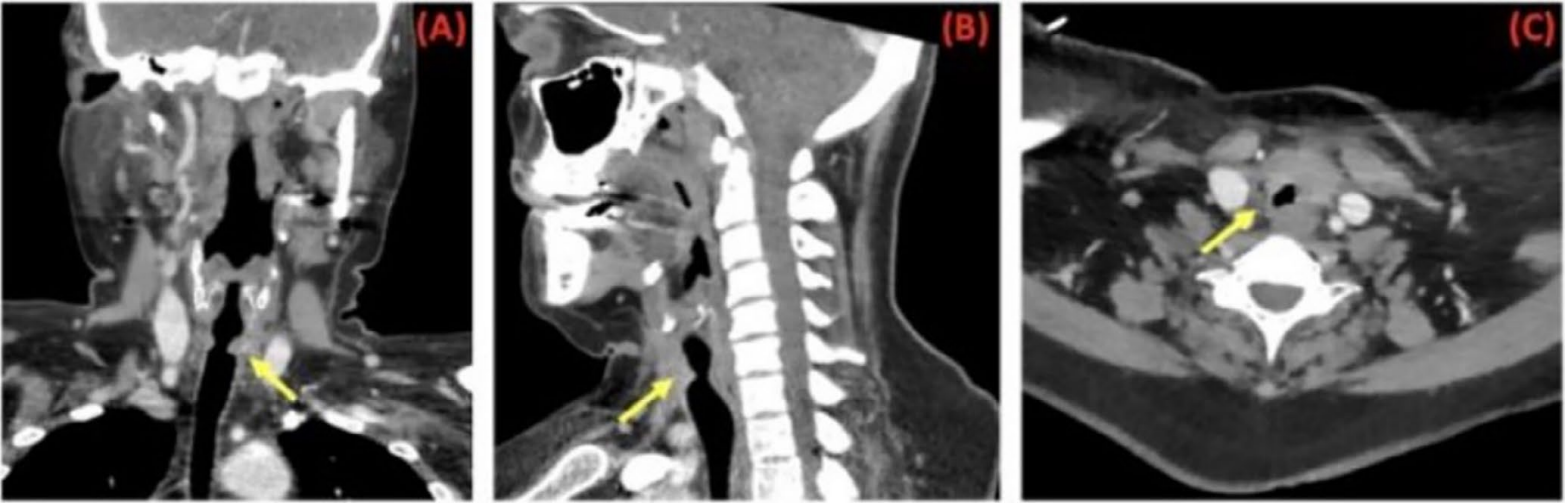
Contrast‐enhanced CT neck and thorax images demonstrating the proximal intraluminal lesion, indicated by yellow arrows: (A) coronal, (B) sagittal, (C) axial views.

Microlaryngobronchoscopy revealed a broad‐based lesion arising from the left superior trachea, occupying approximately 70% of the lumen (Figure 2). The macroscopic appearance was suspicious for a neoplastic process. Partial CO_2_ laser debulking was undertaken to obtain tissue for histopathology and improve airway patency. Preliminary histopathology demonstrated an adenocarcinoma‐pattern neoplasm with immunophenotypic features suggestive of a salivary‐type tumor, including strong diffuse CK7 expression and heterogeneous CK5/6 positivity.

**FIGURE 2:**
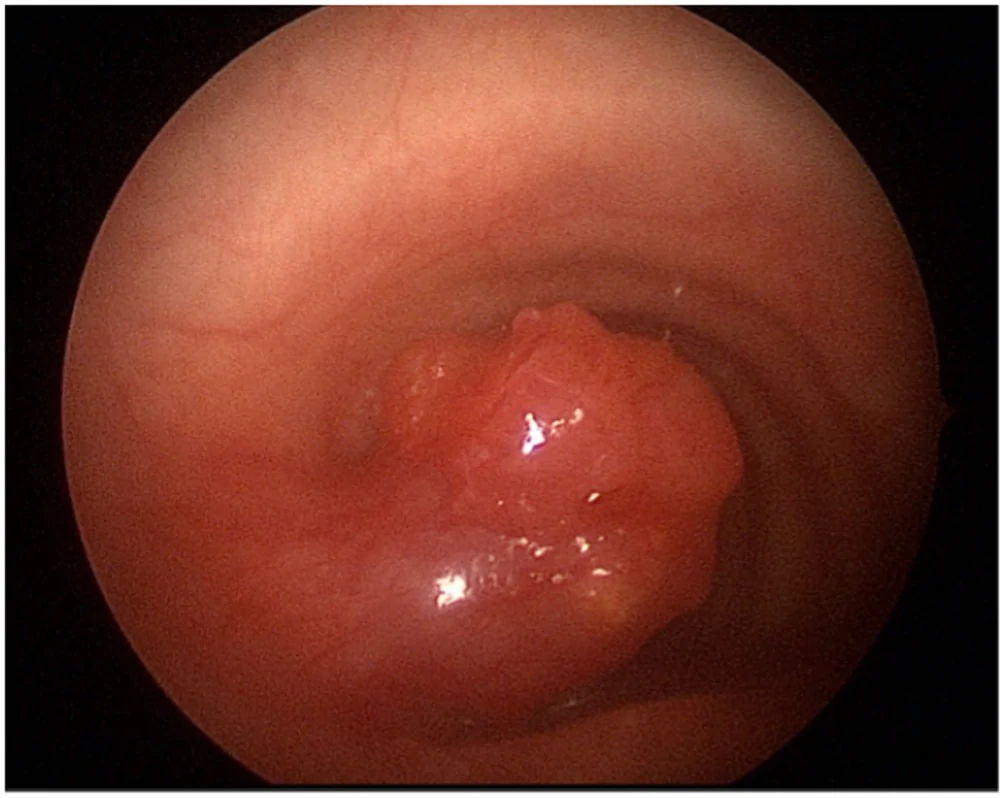
Pre‐operative microlaryngobronchoscopy demonstrating a broad‐based lesion arising from the left wall of the proximal trachea and obstructing approximately 70% of the tracheal lumen.

### 2.4 Therapeutic Intervention

Following multidisciplinary discussion, definitive surgery was undertaken with curative intent. A total thyroidectomy was performed to facilitate surgical access, followed by a segmental tracheal resection and reconstruction with sternothyroid rotatory flaps bilaterally. Deep cartilaginous margins were included due to an intra‐operative clinical concern regarding possible cartilage invasion.

### 2.5 Follow‐Up and Outcomes

Final histopathological examination confirmed a completely excised tracheal MEC with clear margins and no tracheal cartilage invasion. The tumor was classified as low to intermediate grade. It was staged pT1 (8th edition TNM classification). MAML2 rearrangement testing was not performed.

The immediate post‐operative course was initially uncomplicated. Transient hypocalcaemia resolved by postoperative day 3. On postoperative day 5, the patient developed acute dyspnoea associated with left‐sided neck swelling and localized surgical emphysema. This was considered consistent with a tracheal anastomotic leak. The wound clips were partially opened to facilitate decompression, with clinical improvement. She was discharged on postoperative day 15.

On postoperative day 25, she re‐presented with positional stridor. Initial management comprised systemic corticosteroids and nebulised adrenaline. Head and neck examination including flexible nasoendoscopy (FNE) was unremarkable. Rigid bronchoscopy at 6 weeks demonstrated significant anastomotic stenosis characterized by an A‐frame deformity with associated granulation tissue. Biopsy of the stenotic segment demonstrated fibrosis with exuberant, richly vascular granulation tissue and mixed inflammatory infiltrates, consistent with postoperative change, with no definite evidence of disease recurrence.

Endoscopic laser therapy was used to ablate scar and granulation tissue, followed by balloon dilatation to increase the caliber of the stenotic segment. Intralesional corticosteroid was subsequently injected with the aim of reducing the inflammatory response and limiting recurrent scar formation. At 8 weeks postoperatively, the patient reported complete resolution of symptoms. The patient has been asymptomatic and disease‐free on surveillance for 2.5 years.

## 3 Discussion

### 3.1 Diagnostic Considerations

Tracheal MEC often presents insidiously with nonspecific airway symptoms, including cough, exertional dyspnoea, throat‐clearing, globus sensation, or stridor. Similar presentations in adult and pediatric reports have been attributed initially to more common benign airway conditions (such as asthma), contributing to delayed diagnosis [6, 7, 8]. This case exemplifies the diagnostic challenge posed by atypical proximal airway pathology.

Non‐resolving upper‐airway symptoms, including persistent stridor, globus pharyngeus, and throat‐clearing, should therefore prompt cross‐sectional imaging and formal endoscopic assessment when the initial examination does not explain the clinical picture. In this patient, ultrasound appropriately characterized the palpable thyroid abnormality but could not exclude a separate intraluminal airway lesion. CT or MRI can define the site and extent of tracheal lesions, while microlaryngobronchoscopy permits direct assessment, biopsy, and airway intervention [6, 7].

Differential diagnoses for a proximal intraluminal tracheal lesion include adenoid cystic carcinoma, myoepithelial carcinoma, squamous cell carcinoma, papilloma, carcinoid tumor, chondrosarcoma, thyroid cancer with invasion, or benign pathology such as post‐intubation granulation [7, 9, 10], amongst other considerations. Tissue diagnosis and multidisciplinary review are therefore essential before definitive treatment.

### 3.2 Histopathology and Definitive Management

Histological grade, disease extent and completeness of resection are important prognostic determinants in MEC [6]. Pulmonary and tracheobronchial MEC are more frequently reported in the literature. They characteristically contain mucous, epidermoid, and intermediate cells. Immunophenotypic findings and demonstration of MAML2 rearrangement may support the diagnosis, particularly in small or morphologically challenging specimens, but molecular results should be interpreted with the histological and immunohistochemical findings [11].

Management of tracheal MEC is guided by tumor grade, anatomical extent, resectability and margin status. Reported approaches include endoscopic debulking, segmental tracheal resection and adjuvant radiotherapy in selected higher‐grade tumors or when excisional margins are close [6, 12]. In this case, preliminary CK7 and CK5/6 expression supported a salivary‐type neoplasm, while definitive resection established MEC with clear margins. Deep cartilaginous margins were included because of intraoperative concern regarding cartilage involvement, although none was identified histologically.

### 3.3 Post‐Operative Tracheal Stenosis and Endoscopic Management

An important aspect of this case was the early postoperative airway complication. Complications occur in approximately 20% of patients undergoing tracheal resection in larger surgical series, with anastomotic complications including granulation tissue, stenosis, and separation accounting for half of these complications [13]. New dyspnoea, stridor, cough, voice changes, neck swelling, or surgical emphysema after tracheal surgery should therefore prompt reassessment of airway integrity and patency [13].

The patient developed surgical emphysema on postoperative day 5, followed by symptomatic anastomotic stenosis approximately 3 weeks later. The early surgical emphysema was considered consistent with air escape through the healing anastomosis [13], while histological examination of the stenotic tissue demonstrated fibrosis, exuberant vascular granulation tissue and nonspecific inflammation without definite recurrent MEC. Together, these findings support A‐frame deformity and reactive granulation/fibrotic healing as the principal components of the stenosis. Whether the preceding anastomotic leak contributed to subsequent stenosis cannot be established. The extent of tracheal resection, inclusion of deep cartilaginous margins, rotational muscle flap reconstruction, and impaired or delayed tissue healing may also have influenced the outcome, although their respective contributions cannot be determined from a single case.

Rigid bronchoscopy provided both direct assessment and an opportunity for treatment. The combination of modalities was selected to address different components of the stenosis: laser therapy was used to ablate scar and granulation tissue, balloon dilatation provided controlled mechanical expansion of the narrowed segment, and intralesional corticosteroid was administered to reduce the local inflammatory response and subsequent scar re‐formation [13, 14, 15, 16, 17]. The intervention was followed by complete symptomatic resolution, which has been maintained for more than 2.5 years.

### 3.4 Surveillance

Follow‐up after complete resection should consider both disease recurrence and postoperative airway patency. A retrospective cohort of primary tracheobronchial MEC used clinical review and contrast‐enhanced CT every 3 months for the first 2 years, and every 6 months thereafter. Surveillance should be individualized according to risk profile (tumor grade, stage, margins, symptoms) and local practice [18]. In a patient with early postoperative stenosis, recurrence of stridor or dyspnoea should prompt a low threshold for repeat endoscopic assessment. Follow‐up has continued for more than 2.5 years following endoscopic treatment, with sustained clinical resolution of the airway symptoms. The patient remains under regular head and neck follow‐up, currently at approximately six‐monthly intervals, with ongoing imaging surveillance.

### 3.5 Patient Perspective

“From the day the tumur was diagnosed as cancerous, I was scheduled for surgery very quickly. I was otherwise in good physical health and felt that my recovery went well. A few days after surgery I developed surgical emphysema, but the ENT team were available and visited often, and the ward staff were excellent. I am now nearly three years postoperatively and I am in great shape. I continue to see Mr Ahmed twice a year.”

### 3.6 Strengths and Limitations

A strength of this report is the longitudinal description of the diagnostic pathway, curative tracheal resection, recognition of an early anastomotic complication, and its successful multimodal endoscopic management with more than 2.5 years of sustained clinical improvement. A limitation of this report is that MAML2 rearrangement testing was not performed.

### 3.7 Learning Points

- Persistent upper‐airway symptoms and stridor require further investigation when the initial examination does not explain the clinical picture. A normal flexible nasoendoscopy does not exclude a proximal tracheal lesion.
- Recurrent stridor after tracheal resection should prompt urgent assessment for anastomotic or postoperative airway complications.
- In selected early postoperative tracheal stenosis, combined endoscopic laser therapy, balloon dilatation, and intralesional steroid injection may restore airway patency and relieve symptoms.

## 4 Conclusion

Proximal tracheal MEC is a rare malignancy that may remain undetected despite persistent symptoms and a normal initial upper‐airway examination. This case also demonstrates that clinically important stenosis can develop within weeks of complete surgical resection. Prompt recognition of recurrent stridor, direct endoscopic assessment, and morphology‐directed intervention are central to restoring airway patency, with longer‐term oncological and airway surveillance required.

## Author Contributions

**Shivanjali Pandya:** writing – original draft, writing – review and editing. **Gani Nuredini:** writing – original draft, writing – review and editing. **Jahangir Ahmed:** supervision. **Khalid Ghufoor:** supervision.

## Funding

The authors have nothing to report.

## Consent

Written informed consent was obtained from the patient for publication of this case report and the accompanying images.

## Conflicts of Interest

The authors declare no conflicts of interest.

## Data Availability

The data that support the findings of this study are available from the corresponding author upon reasonable request.
